# Solitary Giant Intramuscular Myxoid Neurofibroma Resulting in an above Elbow Amputation

**DOI:** 10.1155/2012/353215

**Published:** 2012-11-03

**Authors:** Gururajaparasad Chennakeshaviah, Sunila Ravishankar, Rangaswamy Maggad, G. V. Manjunath

**Affiliations:** Department of Pathology, J.S.S. Medical College, J.S.S. University, Karnataka, Mysore, 570015, India

## Abstract

Neurofibromas are uncommon benign tumours and are still rarer in intramuscular locations. They are not detected until they cause a significant damage to the neighbouring tissues. We present a case of a giant intramuscular myxoid neurofibroma of the left forearm which eroded the radius and ulna, restricting the movements at the elbow and wrist joints and causing wrist drop resulting in an above elbow amputation. It was diagnosed by histopathology and was later confirmed by immunohistochemistry.

## 1. Introduction

**Figure 1:**
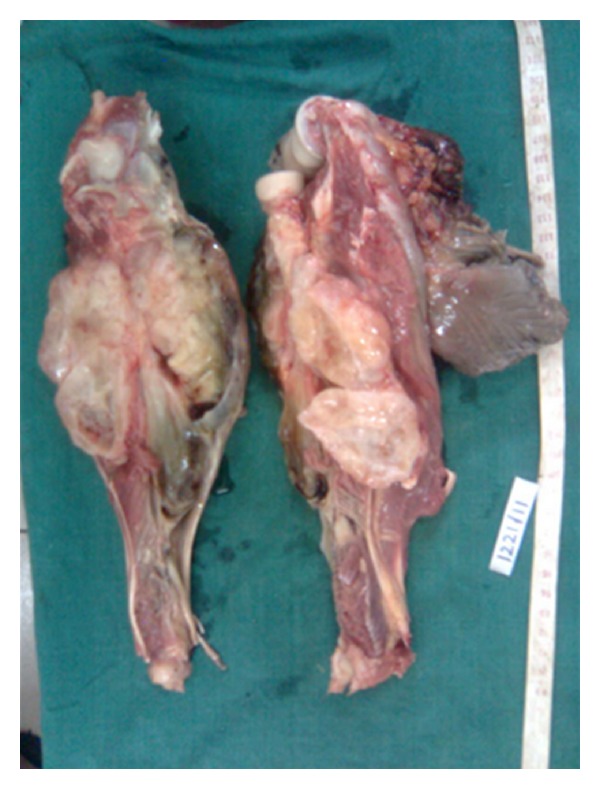
Cut section of the specimen showing a grey white myxoid tumour located intramuscularly between radius and ulna.

Neurofibromas are tumours of the nerve sheath origin and may arise from the Schwann cells, perineural cells, and fibroblasts. Intramuscular neurofibromas are rare [1] and not detected till they cause a significant damage to the neighbouring tissues. We present a case of a giant intramuscular myxoid neurofibroma of the left arm which caused restricted movements of the elbow and wrist joints, erosion of the radius and ulna (Figure 1) and, wristdrop.

We present this case because being benign in nature, due to its giant size and location, this tumour caused complications like wristdrop, erosion of the bones and resulted in an above the elbow amputation. 

## 2. Case Presentation

Here we present a case of 70 yrs male who presented with a painless swelling of the left forearm for the past one year, which incapacitated the movements at the elbow and wrist joints and resulted in wrist drop.

On clinical examination, the tumour was deep seated and the X-ray showed erosion of the radius and ulna. A clinical diagnosis of a malignant soft tissue tumour was made.

The patient was thoroughly examined for the clinical manifestations of neurofibromatosis. There were no “cafe au lait” spots, two or more neurofibromas, Lisch nodules, axillary or inguinal freckling, sphenoid wing dysplasia or thinning of the cortex of long bone, and optic glioma. At least two or more lesions are required for the diagnosis of neurofibroma [1].

 An above elbow-amputated specimen of the left upper limb was received. The dissection showed a glistening white tumour located within the forearm muscles measuring 13.5 × 10.5 cms in association with the median nerve. Cut section was ivory white and glistening with myxoid areas.

## 3. Histopathology: (See Figures 2 and 3)

Histopathological examination showed a tumour with the cells arranged in lobules of anastamosing cords, strands, and nests against a background of myxoid material and intervening fibrous septae. The cells were spindle to elongated with scanty cytoplasm and showed elongated wavy nuclei. No mitoses were seen. Areas of haemorrhage were noted. A diagnosis of myxoid neurofibroma was made.

 Immunohistochemistry showed expression of S100P by the tumour cells.

## 4. Discussion

Neurofibromas (NF) are rare tumours [1] and are further rare in intramuscular locations. All the cases are not associated with neurofibromatosis (NF1). The diffuse and the plexiform patterns have a close relation with neurofibromatosis. The solitary (sporadic) form occurs in those who do not have neurofibromatosis [1]. Myxoid neurofibromas are rarer than the typical ones. These tumours are confused often clinically and histopathologically with myxomas. Myxomas have no nerve involvement and are S100 negative [2].

The other differential diagnoses are the following.

- Aggressive angiomyxoma are perineal or pelvic in location, shows prominent medium to large vessels.
- Low-grade fibromyxoid sarcoma shows alternating fibrous myxoid patterns with swirling and whorled appearance.
- Myxoid liposarcoma shows arborising vascular pattern, signet ring type of lipoblasts, and are more cellular.
- Nodular fasciitis moderately cellular with undulating bundles of cells, tissue culture appearance with tears, and spaces in the tissue.
- Synovial sarcoma with monophasic or biphasic type and myxoid areas.
- Cellular myxoma showing moderate cellularity (20%), arching blood vessels, and collagenous stroma.
- Chordoma which shows physaliphorous cells.
- Schwannoma shows spindle cells with Antony A and Antony B areas.

Histochemical and immunohistochemical stains are useful in the final diagnosis [1].

 Myxoid neurofibroma (MN) is a benign tumour of perineural origin, which is demonstrated by a positive immunohistochemical staining for S100 protein. The most common locations are the face, shoulder, anus, periungal, and in the feet. Only one case occurring in the trunk is reported [2].

The presence of mitotic activity in neurofibroma is indicative of malignancy and is seen even in schwannoma [3].

One case each of a giant genitourinary plexiform neurofibroma associated with lower limb gigantism [4], periungal myxoid neurofibroma [5] in the large bowel [6], a solitary retroperitoneal neurofibroma without any stigmata of Von Recklinghausen's disease [7], in the male breast [8], in the female breast [9], and a case of sacral neurofibroma [10] are reported. This case is being presented because of its rare location, a giant size, erosion of bones ulna and radius which incapacitated the movements at the elbow and wrist joints, resulting in wrist drop and had to end up in an “above elbow amputation”.

## Figures and Tables

**Figure 2:**
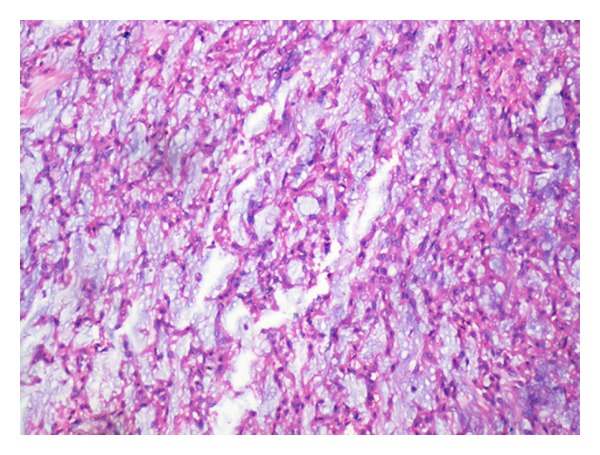
Spindle cells enmeshed in myxoid stroma (H & E ×100).

**Figure 3:**
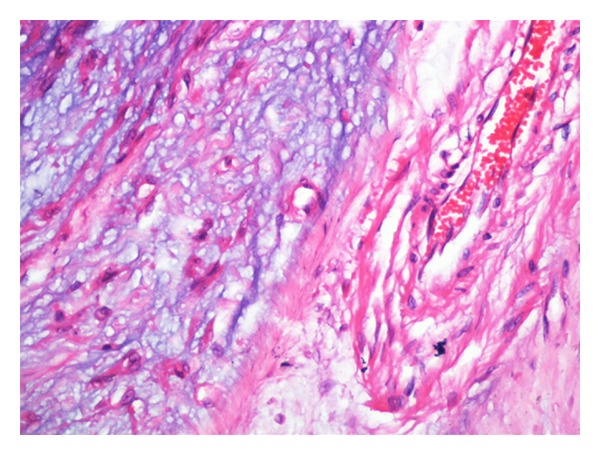
Tumour cells invading the muscles (H & E ×400).
